# Knowledge levels of midwives regarding the interpretation of cardiotocographs at labour units in KwaZulu-Natal public hospitals

**DOI:** 10.4102/curationis.v42i1.2007

**Published:** 2019-11-27

**Authors:** Sindiwe James, Ntsepiseng E. Maduna, David G. Morton

**Affiliations:** 1 Department of Nursing Science, Faculty of Health Sciences, Nelson Mandela University, Port Elizabeth, South Africa

**Keywords:** cardiotocography, cardiotocograph tracings, foetal monitoring, CTG interpretation, intrapartum management

## Abstract

**Background:**

The primary purpose of cardiotocography is to detect early signs of intrapartum hypoxia and improve foetal outcomes. Intrapartum hypoxia remains the major cause of perinatal deaths during monitored labours. This is attributed to the midwives’ lack of knowledge and skills in the foetal implementation and interpretation of cardiotocographs.

**Objectives:**

This study aimed to establish midwives’ knowledge and interpretive skills of cardiotocography.

**Method:**

The study employed a quantitative research approach with an explorative, descriptive, cross-sectional design. A total of 226 purposively selected participants were asked to complete a self-administered, structured questionnaire, of which 125 responded by completing the questionnaire. The study was conducted in labour wards in KwaZulu-Natal public hospitals in 2014. Data analysis was performed by means of descriptive and inferential statistics using analysis of variance.

**Results:**

The findings revealed that the midwives in KwaZulu-Natal public hospitals were found to be clinically lacking in knowledge of cardiotocography.

**Conclusion:**

The limited cardiotocographic knowledge of the midwives in KwaZulu-Natal public hospitals was possibly because of a lack of in-service training, as more than half of the participants (70%) indicated a need for this.

## Introduction

Cardiotocography (CTG) has been acknowledged for decades by obstetric and midwifery practitioners as an effective diagnostic tool during the intrapartum phase. The avoidance of adverse foetal outcomes is the objective of intrapartum foetal monitoring, and thus doctors and midwives often utilise CTG to make diagnoses during the critical time of labour. Therefore, as midwives are the constant caregivers of labouring women, it is fundamental that they have adequate knowledge of CTG to interpret cardiotocographs accurately. This knowledge is necessary for critical decision-making during intrapartum monitoring activities. Despite the long history of CTG surveillance and diagnosis, there are still calls for clinicians to be given stringent training regarding the knowledge and interpretation of cardiotocographs (Ugwumadu et al. 2016:868). This article explores the knowledge levels of midwives practising in labour units in KwaZulu-Natal public hospitals regarding the interpretation of cardiotocographs. Midwives are the focus of the study, as they are the healthcare providers who are almost always present at the bedside of women needing CTG and related interventions.

## Background

Cardiotocography was introduced into obstetric and midwifery clinical practice more than five decades ago on the premise that it would reduce neonatal mortality rates (Fedorka 2010:15). This gave rise to the global use of CTG as a golden standard for intrapartum foetal surveillance (Sartwelle 2012:318). Numerous studies concur that the correct use of CTG reduces the incidence of neonatal seizures associated with cerebral palsy, neonatal as well as perinatal morbidity and mortality (Alfirevic et al. 2017:3; Barstow, Gauer & Jamieson 2010:653; Chen et al. 2011:1); therefore, the value of CTG in maternal and neonatal care is not to be underestimated.

Although CTG was introduced into obstetrics and midwifery clinical practice a long time ago, it has not yet fully lived up to the expectation that it would contribute towards the reduction of perinatal morbidity and mortality (Carbonne & Sabri-Kaci 2016:111). Globally, research findings have shown that the efficacy of CTG has been continuously undermined by the lack of competency of midwives among other healthcare professionals who make use of this intrapartum tool (McKevitt, Gillen & Sinclair 2011:279).

According to Lutomski et al. (2015:3), CTG has some significant challenges related to its interpretation by midwives and doctors. In addition, numerous studies frequently highlight that the level of agreement in the usage and interpretation of the CTG is poor among professionals (McKevitt et al. 2011:279; Santo & Ayres-de-Campos 2012:85). Factors that influence the competency and interpretation of the cardiotocograph are various and diverse. Lutomski et al. (2015:4) affirm that the interpretation of cardiotocograph tracings is subject to individual interpretation and the diagnostic accuracy of midwives and clinicians. However, as Ugwumadu et al. (2016:866) highlight, one of the main causes for litigation in obstetrics is poor cardiotocograph interpretation, suggesting that there is a need for the training of midwives and obstetricians in the knowledge of cardiotocograph interpretation.

The Saving Babies Report presented a summary of the Perinatal Problem Identification Programme (PPIP) findings between 2012 and 2013 and revealed that care assessments were of poor quality in the Eastern Cape, Gauteng and KwaZulu-Natal provinces (Pattinson 2013:26). Prior to this, the Saving Babies Report (2010–2011) indicated that 43% of newborn babies born with a weight of ≥1000 g died of intrapartum hypoxia in South Africa (Pattinson 2013:11). From January 2012 to December 2013, there were 1 412 355 births, with 32 662 stillbirths and 14 576 early neonatal deaths were recorded on the national PPIP database from 588 PPIP (Pattinson & Rhoda 2014:2). According to the most recent report, one of the major causes of death for babies weighing 500 g–1000 g remains intrapartum asphyxia from avoidable factors. For example, in public district hospitals, there were a total of 6082 deaths because of foetal hypoxia (Pattinson & Rhoda 2014:22). A total of 8.4% (*n* = 510) foetuses died of hypoxia that had not been detected during intrapartum monitoring, while 4.6% (*n* = 282) had not been monitored at all. Hence, foetal asphyxia was not detected and the neonates died of foetal hypoxia (Pattinson & Rhoda 2014:26). During the period 2014–2015, 13.43% (*n* = 417) of neonatal deaths occurred when foetal distress went undetected in monitored labours (Rhoda et al. 2018:11).

Against this background, it is therefore evident that there is a need for adequately skilled midwives in South Africa who are equipped to utilise the cardiotocograph machine effectively. A study conducted in the Western Cape in 2011 revealed that midwives lacked knowledge of the use of CTG (Tities 2012:35). There are CTG guidelines available, but these are utilised by a range of healthcare professionals with different skill sets and who may have challenges understanding concepts or in application, and with retaining knowledge over a long-term period (Santo & Ayres-de-Campos 2012:86). Hence, there is a great need for health professionals, specifically midwives, to have the ability and sufficient knowledge of CTG, particularly regarding the application and interpretation of the cardiotocograph.

## Problem statement

In KwaZulu-Natal, midwives often fail to detect signs of foetal compromise during intrapartum foetal monitoring because of their lack of knowledge and skills in CTG. Alfirevic et al. (2017:8) argue that the knowledge and interpretation of CTG tracings vary not only between individual midwives but also between the interpretations made by the same individuals analysing cardiotocograph tracings on successive occasions.

## Aim of the study

The study aimed to explore the knowledge levels of midwives practising in labour units in KwaZulu-Natal public hospitals regarding the interpretation of cardiotocographs.

## Methods

A quantitative research approach was employed with a descriptive cross-sectional design (Houser 2012:286). The population chosen for this study was all the midwives working in labour and delivery units in the public hospitals of KwaZulu-Natal. The sampling frame (Hedges & Williams 2014:298) was drawn from nine public hospitals and consisted of 241 midwives. Following a purposive criterion-based sampling method, 226 participants were found to be suitable for the study. All the midwives were targeted for sampling purposes, with the exception of midwives holding managerial positions and those awaiting disciplinary hearings. The inclusion criteria included having clinical experience of at least 2 years as a qualified professional nurse and midwife as well as having been assigned full time in the labour wards of the selected public hospitals for at least 2 years.

Participants were asked to complete a structured and self-administered questionnaire. A self-administered questionnaire is a printed self-report form designed to elicit information through written responses on the subject (Grove, Burns & Gray 2013:45; Polit & Beck 2018:168). The questionnaire comprised four sections with closed and open-ended questions. Open-ended questions were included to enable the participants to give their own unique, individual opinions. The questionnaire was divided into the following sections: biographical and educational data, participants’ perceptions regarding their levels of competence related to CTG tasks, knowledge of foetal monitoring, and a CTG interpretational skills test. The last section of the questionnaire required the participants to view a CTG strip, respond to set questions and compare the responses with those in Section B.

The questionnaire was examined for face and content validity by independent experts and a professional statistician who evaluated it for conceptual and investigative bias. The instrument was also submitted to a review panel including lecturers from an Advanced Life Support in Obstetrics programme and two foetal medicine specialists, who are considered to be experts in electronic foetal monitoring. The panel was requested to indicate its agreement with the scope of the items and the extent to which they reflected the concept under consideration (Polit & Beck 2018:176). Furthermore, midwifery labour and delivery unit managers from outside the KwaZulu-Natal province were requested to review the questionnaire for face and content validity. Thus, the reliability of the questionnaire was ensured by the involvement of midwifery experts in the reviewing process. Moreover, 1 week before the main study, reliability was enhanced through a pilot study involving 10 participants in one hospital who were not part of the sample.

## Data collection

The research was conducted over a 5-month period in nine public hospitals that were purposively selected in KwaZulu-Natal. Permission to access the sites was obtained from the participating district hospitals before commencing data collection. Participation was preceded by a session with the managers of each hospital to explain and ask permission to enter and collect data from the midwives. Midwives were informed in each site by means of a written letter that was circulated through the hospital managers’ offices. After information sessions, voluntary participants gave written informed consent, although they also knew that they were free to withdraw their participation at any stage of the study without being penalised. Altogether, 125 participants returned the completed questionnaires, yielding a response rate of 55%. This was somewhat low with Gerrish and Lacey (2010:378), stating that a response rate of more than 75% is considered to be a good response rate. Codes were used for the questionnaires to ensure privacy, anonymity and confidentiality of data to which only the researchers and the statistician had access. Completed questionnaires were handled only for the purpose of data analysis and were otherwise stored under lock and key.

### Ethical considerations

Ethics approval was obtained from the Nelson Mandela University ethics committee (ethics clearance number: H13-HEA-NUR-018).

## Results

Data were captured and entered into an Excel spreadsheet for the purpose of data analysis according to a framework developed by statisticians. The data were analysed using analysis of variance (ANOVA) and *t*-tests to determine the correlation between the demographic profiles of the participants and their levels of CTG knowledge regarding the interpretation of the cardiotocograph.

### Demographic profile

The questionnaire contained a section addressing the demographic profile of the participants. It focused on age; clinical experience in midwifery; labour ward experience; competence level with regard to CTG tasks additional post-basic midwifery qualification; and CTG in-service education. The results of the demographic profile are presented in Table 1.

**TABLE 1:** Demographic profile of midwives participants.

Participants (*n* = 125)	Variable	*n*	%
Age (year)	21–30	17	14
	31–40	44	35
	41–49	30	24
	50 and above	34	27
Clinical midwifery experience (years)	2	18	14
	3–5	15	12
	6–10	23	29
	11–20	34	27
	21 and above	32	18
Labour ward experience (years)	2	23	18
	3–5	30	24
	6–10	28	22
	11–20	35	28
	21 and above	9	7
Post-basic additional midwifery qualification	Yes	66	53
	No	59	47
Last in-service education on CTG (years)	˂ 1	62	50
	1–4	33	27
	5 and above	29	23

CTG, cardiotocography.

### Age and experience

Thirty-five per cent (*n* = 44) of the participants were between 31 and 40 years old and constituted the bulk of the sample. Participants between 21 and 30 years old made up the smallest proportion (14%, *n* = 18) of the participants. The majority of the participants (56%, *n* = 125) reported their clinical midwifery experience as being from 6 to 20 years. A high percentage (50%, *n* = 72) of the participants had worked between 6 and 20 years in labour wards. Few participants (7%, *n* = 9) reported working in labour wards for more than 20 years.

### Education and training

The majority (53%; *n* = 66) of the participants indicated that they possessed an additional post-basic midwifery qualification; and (89%; *n* = 59) had a post-basic diploma in Advanced Midwifery and Neonatal Nursing Science. Only 2% (*n* = 1) of participants possessed a master’s degree. Altogether 50% (*n* = 62) reported that they had last received in-service education on CTG less than a year before. The remaining 50% (*n* = 63) of the participants reported that they had last attended in-service CTG education between 1 and 5 years and above before.

### Knowledge of cardiotocography

The questionnaire focused on establishing the participants’ knowledge of CTG. The midwives were asked questions regarding their knowledge of the following: (1) CTG monitoring, (2) maternal heart rate (MHR) and foetal heart rate (FHR) confusion, (3) CTG labelling, (4) CTG paper speed, (5) maternal and foetal physiology, (6) define risk, contractions, baseline rate, variability, accelerations, decelerations, overall impression (DR C BRAVADO) and (7) CTG interpretation. Table 2 shows the statistical analysis of the midwives’ knowledge of CTG.

**TABLE 2:** Frequency distribution: Percentages of participants’ correct responses to knowledge questions regarding cardiotocography (*n* = 125).

Variable	Score range (0% – 20%)		Score range (20% – 40%)		Score range (40% – 60%)		Score range (60% – 80%)		Score range (80% – 100%)	
	*n*	%	*n*	%	*n*	%	*n*	%	*n*	%
1. CTG monitoring	29	23	22	18	0	0	37	30	37	30
2. MHR and FHR confusion	93	74	0	0	21	17	0	0	11	9
3. CTG labelling	7	6	10	8	2	2	7	6	99	79
4. CTG paper speed	15	13	0	0	0	0	0	0	104	87
5. Maternal and foetal physiology	0	0	6	5	28	22	61	49	30	24
6. DR C BRAVADO	51	41	1	1	3	2	6	5	64	51
7. CTG interpretation	1	1	12	10	46	37	53	42	13	11

CTG, cardiotocography; MHR, maternal heart rate; FHR, foetal heart rate; DR C BRAVADO, define risk, contractions, baseline rate, variability, accelerations, decelerations, overall impression.

### The relationship between the midwives’ demographic profiles and cardiotocography knowledge of midwives

The relationship between the demographic profile and the midwives’ knowledge of FHR and monitoring was explored to determine factors that influenced the midwives’ CTG competency. Analysis of variance and *t*-tests were used to analyse the data. Relationships were explored between CTG knowledge and midwifery clinical experience; CTG in-service education and CTG knowledge; and additional post-basic midwifery qualification and CTG knowledge. The results are presented in Tables 2–5.

Table 3 presents the findings concerning the relationship between midwifery clinical experience and CTG knowledge. A statistically and practically significant difference was found between the mean scores of participants with midwifery clinical experience of between 6 and 10 years, on the one hand, and those of the group with more than 10 years’ experience in labelling the CTG paper, on the other hand. Moreover, a statistically and practically significant difference was found between the means of the group of participants with fewer than 6 years of midwifery clinical experience and the group with more than 10 years of midwifery clinical experience of reducing maternal and FHR confusion.

**TABLE 3:** Midwifery clinical experience and cardiotocography knowledge of participants (*n* = 125).

Variable	Group 1 < 6 years (*n* = 53)		Group 2 6–10 years (*n* = 28)		Group 3 >10 years (*n* = 44)	
	Mean	s.d.	Mean	s.d.	Mean	s.d.
1. CTG monitoring	48.45	37.95	60.75	37.53	59.91	37.81
2. MHR and FHR confusion	17.92	35.48	16.07	27.40	17.05	30.39
3. CTG labelling	79.62	34.53	77.86	34.14	89.55	23.42
4. CTG paper speed	84.43	36.14	96.43	18.90	84.09	37.00
5. Maternal and foetal physiology	67.40	14.37	65.86	16.49	64.73	15.00
6. DR C BRAVADO	51.81	46.28	52.57	44.71	53.32	45.05
7. CTG interpretation	58.35	18.02	61.59	13.74	61.44	17.70

CTG, cardiotocography; MHR, maternal heart rate; FHR, foetal heart rate; s.d., standard deviation; DR C BRAVADO, define risk, contractions, baseline rate, variability, accelerations, decelerations, overall impression.

The study results revealed that there was no significant difference in the scores obtained by participants based on their last in-service education on CTG with the *p*-value of 0.863, as shown in Table 4. Therefore, it was concluded that in-service education did not improve their CTG monitoring; their ability to distinguish between MHR and FHR; their CTG labelling; their understanding of CTG paper speed; their understanding of maternal and foetal physiology; their understanding of DR C BRAVADO; and their CTG interpretation.

**TABLE 4:** Cardiotocography in-service education and cardiotocography knowledge of participants (*n* = 124).

Variable	Group 1 < 1 year ago (*n* = 62)		Group 2 1–4 years ago (*n* = 33)		Group 3 5+ years ago (*n* = 29)	
	Mean	s.d.	Mean	s.d.	Mean	s.d.
1. CTG monitoring	52.24	35.60	55.55	42.27	60.90	38.97
2. MHR and FHR confusion	12.10	25.10	15.15	31.83	31.03	41.00
3. CTG labelling	80.65	31.77	84.24	30.72	84.83	31.47
4. CTG paper speed	91.94	27.45	81.82	39.17	82.76	38.44
4. Maternal and foetal physiology	66.52	14.33	64.24	16.40	68.28	14.46
6. DR C BRAVADO	50.98	44.00	61.09	45.24	47.83	47.46
7. CTG interpretation	59.07	16.40	60.35	16.80	62.60	18.89

CTG, cardiotocography; MHR, maternal heart rate; FHR, foetal heart rate; s.d., standard deviation; DR C BRAVADO, define risk, contractions, baseline rate, variability, accelerations, decelerations, overall impression.

**TABLE 5:** Additional post-basic midwifery qualification and cardiotocography knowledge (*n* = 125).

Variable	Additional qualification	*N*	Mean	s.d.	Difference	*T*	df	*p*	*d*
1. CTG monitoring	Yes	66	66.73	34.14	24.34	3.76	123	< 0.0005	0.67
	No	59	42.39	38.14	-	-	-	-	Medium
2. MHR and FHR confusion	Yes	66	20.45	35.06	6.90	1.21	123	0.228	0.22
	No	59	13.56	27.59	-	-	-	-	Small
3. CTG labelling	Yes	66	86.36	28.32	7.72	1.39	123	0.167	0.25
	No	59	84.75	33.81	-	-	-	-	Small
4. CTG paper speed	Yes	66	89.39	31.03	4.65	0.77	123	0.441	0.14
	No	59	84.74	36.26	-	-	-	-	Not
5. Maternal and foetal physiology	Yes	66	68.12	16.21	4.26	1.59	123	0.114	0.29
	No	59	63.86	13.31	-	-	-	-	Small
6. DR C BRAVADO	Yes	66	57.18	43.93	9.89	1.23	123	0.223	0.22
	No	59	47.29	46.26	-	-	-	-	Small
7. CTG interpretation	Yes	66	64.71	17.91	9.63	3.29	123	0.001	0.59
	No	59	55.08	14.39	-	-	-	-	Medium

CTG, cardiotocography; MHR, maternal heart rate; FHR, foetal heart rate; s.d., standard deviation; DR C BRAVADO, define risk, contractions, baseline rate, variability, accelerations, decelerations, overall impression.

The study findings revealed a small, but statistically and practically, significant difference in the CTG knowledge of participants who had an additional post-basic midwifery qualification, compared to that of those who did not. The participants who held additional post-basic midwifery qualifications had a higher mean score of 66.73 for questions regarding the monitoring of CTG, while their counterparts had a mean score of 42.39, *p* < 0.0005, *d* = 0.67. Another small difference was observed in the mean scores of the groups on CTG interpretation *p* = 0.001, *d* = 0.59. The differences observed between the scores were either small or medium, but were not significant enough to make any inferences. Therefore, it was concluded that there was no significant relationship between the level of education and the CTG interpretation skills of the midwives (*p* = 0.25 and *d* = 0.41 small).

## Discussion

The findings showed that the scores obtained on the interpretation of CTG strips were low, suggesting that the midwives were not strong in this aspect of CTG. This is a matter of concern for clinical practice because the accurate interpretation of CTG is critical, as a misinterpretation of FHR tracings can lead to poor clinical decision-making (Lutomski et al. 2015:3). It was also evident that participants knew FHR monitoring, but could not interpret the findings of the CTG graphs, which is a problem in clinical practice. The 2012–2013 PPIP report reveals that neonatal deaths may have been because of the incorrect use of CTG or even a failure to use it at all for intrapartum monitoring (Pattinson & Rhoda 2014:22, 26).

Numerous studies have documented that in-service CTG training significantly improves a midwife’s expertise of CTG practice (Oleiwi & Abbas 2015:40; Rosie & Princy 2015:43; Sowmya, Priya & Jothi 2013:91). However, this study reveals that the midwives who had recent CTG in-service education did not demonstrate the expected higher level of CTG competence than that of their counterparts who had not been trained. Thus, there is cause for concern regarding the scope and nature of the CTG in-service education that was provided to the participants who appeared to lack the necessary knowledge required to conduct CTG.

The participants’ demography was expected to influence the knowledge of CTG in this study that was underpinned by Benner’s framework as described in ‘From Novice to Expert Model’. According to Benner’s model, competency comes through education and training. Clinical knowledge and experience are gained over time and nurses (midwives) themselves are often not aware of their gains (Health Research Funding 2019). The participants of this study were very experienced and thus expected to have adequate CTG knowledge. In fact, the majority of the participants (89%) were advanced trained midwives. Statistically, 45% (*n* = 66) had 11–21 years of clinical experience, and 35% (*n* = 44) had 11–21 years of working experience in labour wards. According to the South African Nursing Council, in terms of the provision of the *Nursing Act* (No 33 of 2005), an advanced midwife is a highly skilled and competent specialist with sufficient in-depth knowledge and expertise in midwifery to provide quality care to patients during pregnancy, labour and puerperium (Mulondo, Khoza & Risenga 2013:5). Therefore, it was expected that the participants would demonstrate a high level of FHR monitoring expertise. However, these expectations were not met by the statistical results that emerged from this study. Moreover, the study findings provide evidence that the perceived level of competence in CTG tasks did not positively influence the low level of FHR monitoring knowledge demonstrated by the participants. At least 70% of participants reported that they were competent, yet they practically demonstrated knowledge deficits in FHR monitoring that is fundamental to CTG interpretation, a skill they also lacked.

Generally, the results revealed that the majority of participants had a certain degree of knowledge in some areas of CTG. For example, participants obtained high scores on the labelling of the CTG paper and the CTG paper speed of the monitor. Nevertheless, the results highlighted a critical knowledge deficit with regard to the monitoring of CTG; the reduction of the risk of foetal and MHR confusion, maternal and foetal physiology; and the mnemonic ‘DR C BRAVADO’. A knowledge deficit regarding the monitoring of CTG is crucial, as it implies that the midwives are not performing a CTG where it is required and it further casts doubt on their ability to interpret cardiotocographs and utilise CTG. Furthermore, MHR could be misinterpreted as foetal compromise, and thus, result in unnecessary interventions. The ‘DR C BRAVADO’ mnemonic device provides a systematic approach to CTG interpretation, which is fundamental to clinical decision-making during FHR monitoring. However, if this approach is not followed, there is a risk of misinterpreting CTGs. A lack of knowledge of foetal physiology affects the ability of midwives to detect signs of foetal hypoxia during intrapartum surveillance. This knowledge deficit, if not addressed, can have serious consequences on foetal outcome. Moreover, litigation might follow because of this incapacity (Santo & Ayres-de-Campos 2012:84). Consequently, Alfirevic et al. (2017:9) argue that although CTG is associated with limiting neonatal seizures and hypoxia to a lesser extent, it has also been used as an intrapartum diagnostic tool, which includes the diagnosis of asphyxia (Chudacek et al. 2014:2; Hastings 2015:166). Midwives who are challenged with interpretation of CTG may not be able to make the required decisions and perform the much-needed actions as indicated by the results on the CTG record.

The risk and consequences of MHR and FHR confusion during electronic foetal monitoring have been documented in numerous studies (Behar et al. 2016:10). Furthermore, knowledge of maternal and foetal physiology is fundamental to adequate interpretation of CTG and its related interventions. Pinas and Chandraharan (2016:33) concur that clinicians need to understand the physiology behind the FHR changes and intervene accordingly. The midwives described in this study lacked knowledge of maternal and foetal physiology in relation to CTG. Fifty-two per cent of the participants affirmed that their colleagues could perform CTG efficiently. However, a significant number (59%) of participants reported that they had noticed inconsistencies and disagreements in the interpretation of CTG among their colleagues. Some midwives (20%) agreed that they had misinterpreted a CTG result in the past, and this had led to an intervention. Indeed, Carbonne and Sabri-Kaci (2016:111) indicate that foetal deaths are often because of poor FHR analysis, a lack of identification of pathologic tracings and incorrect or slow response to a pathologic FHR on the part of obstetricians and midwives. Hence, incomplete knowledge of CTG among midwives is a matter of concern.

In the context of this study, a lack of CTG knowledge among practising midwives impacts on their ability to detect signs of foetal hypoxia during intrapartum surveillance. Such a limitation could be costly to the hospital and government, as mothers and families might instigate costly litigation because of the death or disability of the neonate or child. Existing literature attests to the challenge of CTG knowledge, and the results of the study discussed in this article confirm this as well. Thus, the improvement of clinical practice requires a consideration of the different variables that could be barriers to efficient and sufficient CTG knowledge.

### Limitations

The study was conducted in only 4 out of the 11 health districts of KwaZulu-Natal province, and only 9 out of 15 available hospitals actually participated in the study. Therefore, the study results cannot be generalised to the whole of the KwaZulu-Natal province. The data collection process was hindered by the slow return rates of questionnaires because of permission and access challenges and the availability of participants, thus delaying the data collection target date by 3 months. Of the 226 midwives who initially consented to participate in the study, only 125 returned completed questionnaires after an additional area of data collection was approved (a response rate of 55%).

### Recommendations

Midwives need regular CTG in-service training for effective and confident practice. Hence, there is a need to consider the training given to midwives as well as the different approaches to this regarding the various aspects of CTG. Furthermore, the content and frequency of in-service training needs to be reviewed because of its influence on midwives’ knowledge and skills, which, in turn, affects the quality of perinatal care.

Furthermore, there also appears to be a need for national standards to be developed to assist existing evidence-based guidelines for effective FHR monitoring. In the clinical environment, nurse managers are required to be sensitive to such differences. There appears to be a need for the development of set principles regarding CTG knowledge and skills that are taught in midwifery training schools. Furthermore, these should be emphasised at clinical level to enhance competence and effectively integrate the theory and practice that are fundamental to the improvement of midwives’ knowledge of CTG. Managers should promote self-study and group learning to improve knowledge and clinical skills.

Regarding future research, there is a need for intervention studies where in-service training programmes can be implemented and assessed for their effectiveness. In addition, research utilising structured observations during CTG monitoring and cardiotocograph interpretation by midwives is recommended to assess the skills of the midwives. A comparative study between public and private hospitals regarding CTG usage by midwives would help to determine the variations in CTG knowledge and practices across various healthcare settings.

## Conclusion

Cardiotocography knowledge remains a challenge for practising midwives in South Africa. The study findings show that midwives lack knowledge regarding CTG interpretation. The limited CTG knowledge of the midwives in KwaZulu-Natal public hospitals was possibly because of a lack of in-service training, as more than half of the participants (70%) indicated a need for in-service training. Clinical experience and prolonged exposure to regular use of CTG in labour wards did not appear to have a positive influence on the knowledge levels of the midwives. The interpretation and management of CTG is a complex task that requires a sound knowledge of FHR patterns, foetal physiology and intrapartum management, as it is applied to the specific clinical needs of each patient.
